# From single ligament to multi-ligament injury: a finite element study on the contribution of the posterior ligamentous complex to segmental stability and intervertebral disc stress distribution

**DOI:** 10.1186/s12891-025-09110-z

**Published:** 2025-08-25

**Authors:** Jingbo Ma, Yu Ding, Rigbat Rozi, Jiaheng Han, Qiang Jiang

**Affiliations:** 1 Orthopedics of TCM Senior Department, The Sixth Medical Center of People’s Liberation Army General Hospital, Beijing, 100048 China; 2 Navy Clinical College, Fifth School of Clinical Medicine, Anhui Medical University, Hefei, China

**Keywords:** Lumbar spine, Posterior ligamentous complex, Finite element modeling, Stepwise reduction, Inter-subject variability

## Abstract

**Objective:**

The posterior ligamentous complex (PLC) plays a crucial role in maintaining lumbar spine stability. PLC injuries have become a key factor in lumbar instability, with the increase in degenerative spinal conditions and surgical interventions. This study aimed to systematically quantify the impact of single and multi-ligament injuries on spinal stability and analyze their effects on lumbar biomechanical indices and intervertebral disc stress distribution.

**Methods:**

Finite element analysis (FEA) and experimental measurements were employed to examine the effects of 12 ligament resection combinations on lumbar range of motion (RoM) and intervertebral disc stress distribution. Detailed statistical analysis, including the Kruskal-Wallis Test, was used to evaluate the significance of observed differences. Functional contributions of individual ligaments and their combinations were analyzed to assess their roles in restricting spinal motion.

**Results:**

The results indicated that ligament resection combinations significantly impacted lumbar biomechanical indices (*P* = 0.016), with an effect size (η²) of 0.058, reflecting a moderate impact on segmental stability. The interspinous ligament (ISL) demonstrated the most significant role in restricting excessive spinal motion, followed by the ligamentum flavum (LF), while the supraspinous ligament (SSL) and facet joint capsules (FJC) had limited effects. Combined multi-ligament injuries, particularly ISL and LF resection, markedly increased spinal instability and altered intervertebral disc stress distribution. Despite significant stability loss from multi-ligament injuries, intact ligaments provided functional compensation, mitigating instability.

**Conclusion:**

This study revealed the nonlinear cumulative effects of PLC damage on spinal stability, emphasizing the dominant roles of ISL and LF in maintaining biomechanical integrity. The findings provide critical quantitative insights for clinical decision-making, surgical planning, and postoperative rehabilitation strategies, highlighting the importance of preserving intact ligaments to leverage their compensatory capacity in mitigating instability.

## Introduction

The posterior ligamentous complex (PLC) is essential for maintaining the biomechanical stability of spinal segments, particularly in the lumbar spine. It is composed of four key components: the supraspinous ligament (SSL), interspinous ligament (ISL), ligamentum flavum (LF), and facet joint capsules (FJCs) [1]. Under normal physiological conditions, these ligaments function synergistically to limit excessive flexion, extension, and rotation between vertebral bodies, thus ensuring spinal stability during weight-bearing activities and movement [2]. With the aging of the population, there has been a significant increase in the prevalence of degenerative spinal disorders, trauma, and surgical interventions such as posterior decompression and spinal fusion. Consequently, issues related to PLC injuries and structural compromise have grown prominence [3]. Clinically, these injuries may result in lumbar segmental instability, leading to symptoms such as lower back pain, neural compression, and accelerated intervertebral disc degeneration. These conditions significantly influence the design of surgical interventions and postoperative rehabilitation strategies. Investigating how changes in PLC integrity affect lumbar segment stability is not only a fundamental question in medical biomechanics but also provides essential guidance for clinical decision-making, such as whether to preserve or reconstruct posterior structures [4].

Recent studies have underscored the critical role of the PLC in maintaining spinal stability, with numerous experiments and investigations focusing on its biomechanical properties and functions [5]. Traditional research methods, such as cadaveric studies and radiological assessments, are challenged by issues of quantification, parameter adjustment, and reproducibility. Advantageously, advancements in computational mechanics and medical imaging technologies have made finite element analysis (FEA) a more precise tool for investigating the impact of PLC integrity on spinal stability [6]. Recent research using FEA and experimental testing have investigated the mechanical behavior of the spine and its associated structures from various perspectives, emphasizing the critical role of passive structures-such as ligaments, intervertebral discs, and facet joints-in maintaining spinal stability [7]. These evidence indicate that even a single ligament injury can substantially alter stress distribution and range of motion (RoM), compromising overall spinal stability. This suggests that if the nonlinear supportive function of a specific ligament is compromised, the corresponding spinal segment may exhibit increased (RoM) and uneven stress distribution. These changes may ultimately lead to instability and exacerbate degenerative changes in the lumbar spine [8].

However, current literature primarily focuses on the effects of single-ligament injuries or complete PLC disruption on spinal stability, while systematic evaluations of the quantitative differences and cumulative effects of various ligament injury patterns are still insufficient [9]. Previous experimental studies have systematically demonstrated how stepwise resection of posterior ligamentous structures can significantly increase vertebral translation and intradiscal pressure, underscoring the critical biomechanical role of each ligamentous component within the lumbar spine [10]. It remains unclear how segmental stability metrics, such as changes in the RoM and stress concentration patterns, exhibit continuous and quantifiable trends during the progression from partial injury of a single ligament to the combined failure of multiple ligaments [11]. To address these issues, we aim to elucidate the impact of progressive structural degradation, from single ligament to multi-ligament injury, on the stability parameters of the lumbar segment. We hypothesize that the removal of different ligaments will have varying effects on the biomechanical stability of lumbar segments and the stress distribution in intervertebral discs. Additionally, combined multi-ligament resection is expected to produce synergistic effects, leading to a significant decrease in stability and a redistribution of disc stress, thereby clearly demonstrating the nonlinear cumulative effects of ligament injuries on spinal segment stability. Furthermore, considering the functional redundancy of PLC components, we hypothesize that, in case of multi-ligament injuries, the remaining intact ligaments may partially compensate, thereby mitigating the impact of a single-ligament injury on overall stability.

To test this hypothesis, the present study compared the mechanical responses of the entire lumbar segment under three conditions: an intact posterior ligamentous complex, partial resection of a single ligament, and combined resection of multiple ligaments. The study systematically simulated changes in PLC integrity and conducted rigorous data analysis with statistical comparisons to quantitatively assess the contributions of individual PLC components and their combined effects on spinal stability. Different with traditional studies, that primarily focused on single-ligament injuries or relied on simpler methods, this study adopted a more comprehensive and systematic approach to analyze the contribution of each ligament within the PLC to segmental stability. The results are expected to reveal the cumulative effects of progressive ligament damage on key metrics, such as RoM and stress distribution, thereby enhancing our understanding of how different combinations of ligament injuries affect overall spinal stability. Furthermore, this study also aims to provide clinicians with quantitative evidence to inform surgical planning, particularly regarding the preservation of PLC structures [12]. By addressing gaps in existing research, this study offers practical insights into the role of PLC components in maintaining spinal stability, aiming to support better clinical outcomes and advance biomechanical research [13].

## Materials and methods

### Model development

The lumbar spine segment of a 31-year-old healthy male volunteer was chosen as the prototype for this study. This study was approved by the Ethics Committee of the Sixth Medical Center of the General Hospital of the Chinese People’s Liberation Army (No. HZKY-PJ-2025-1). Since our study was not a clinical trial, clinical trial registration was not applicable. High-resolution CT scans were used to perform three-dimensional reconstructions of the vertebral bodies, intervertebral discs, and posterior joint structures. The PLC was modeled as a bilinear elastic structure based on thin-slice magnetic resonance imaging (MRI) of the volunteer. Although initial pre-strain was not explicitly included, the bilinear response effectively approximates in vivo behavior under physiological loading, as supported by prior validated finite element studies [9, 14]. The final geometric model included the upper and lower vertebral bodies, intervertebral discs (composed of endplates, annulus fibrosus, and nucleus pulposus), ligaments, and facet joints. A parametric finite element model of the L1-L5 motion segment was developed using ANSYS (ANSYS, Inc., License Manager 2023 R1) [15]. The solid model was assigned material properties, including Young’s modulus and Poisson’s ratio. Detailed descriptions of the model and parameters are provided in previous publications [16].

### Material properties and contact definitions

The material properties of each component were assigned based on reference data, as summarized in Table 1. [17–20]. The vertebral bodies and posterior bony structures were modeled with isotropic elastic material properties. The intervertebral discs were represented by a composite model comprising the annulus fibrosus, nucleus pulposus, and endplates. No explicit collagen fibers were defined within the intervertebral discs; the annulus fibrosus was modeled as an anisotropic material to represent its layered structure, and the nucleus pulposus was treated as a nearly incompressible material. Facet joints were modeled using frictional contact, with a low coefficient of friction to allow limited relative sliding between joint surfaces.

**Table 1:**
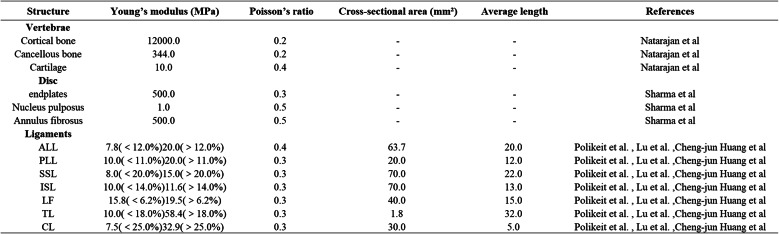
Material properties in the present FE models

FE: finite element; ALL: anterior longitudinal ligament; PLL: posterior longitudinal ligament; SSL: supraspinal ligament; ISL: interspinous ligament; LF: ligamentum flavum; TL: transverse ligaments; CL: capsular ligament; FJC: facet joint capsule

### Definition and simulation of PLC injuries

To investigate the impact of ligament integrity on spinal stability, the L4-L5 segment was selected for ligament injury simulation in this study. The L4-L5 segment is the most mobile pair in the lumbar spine, bearing significant biomechanical loads, making it highly representative in studies of spinal motion and stability. Additionally, the L4-L5 segment is a common site of pathology in the lumbar spine, frequently requiring surgical intervention. Consequently, this study established the following model conditions:

#### Intact condition

All ligaments were preserved with their normal material properties.

#### Single-ligament resection condition

A specific ligament was selected and completely resected to simulate its total failure. This resection involved removing all finite elements corresponding to the ligament in the model, thereby eliminating any biomechanical support from the ligament.

**Fig. 1:**
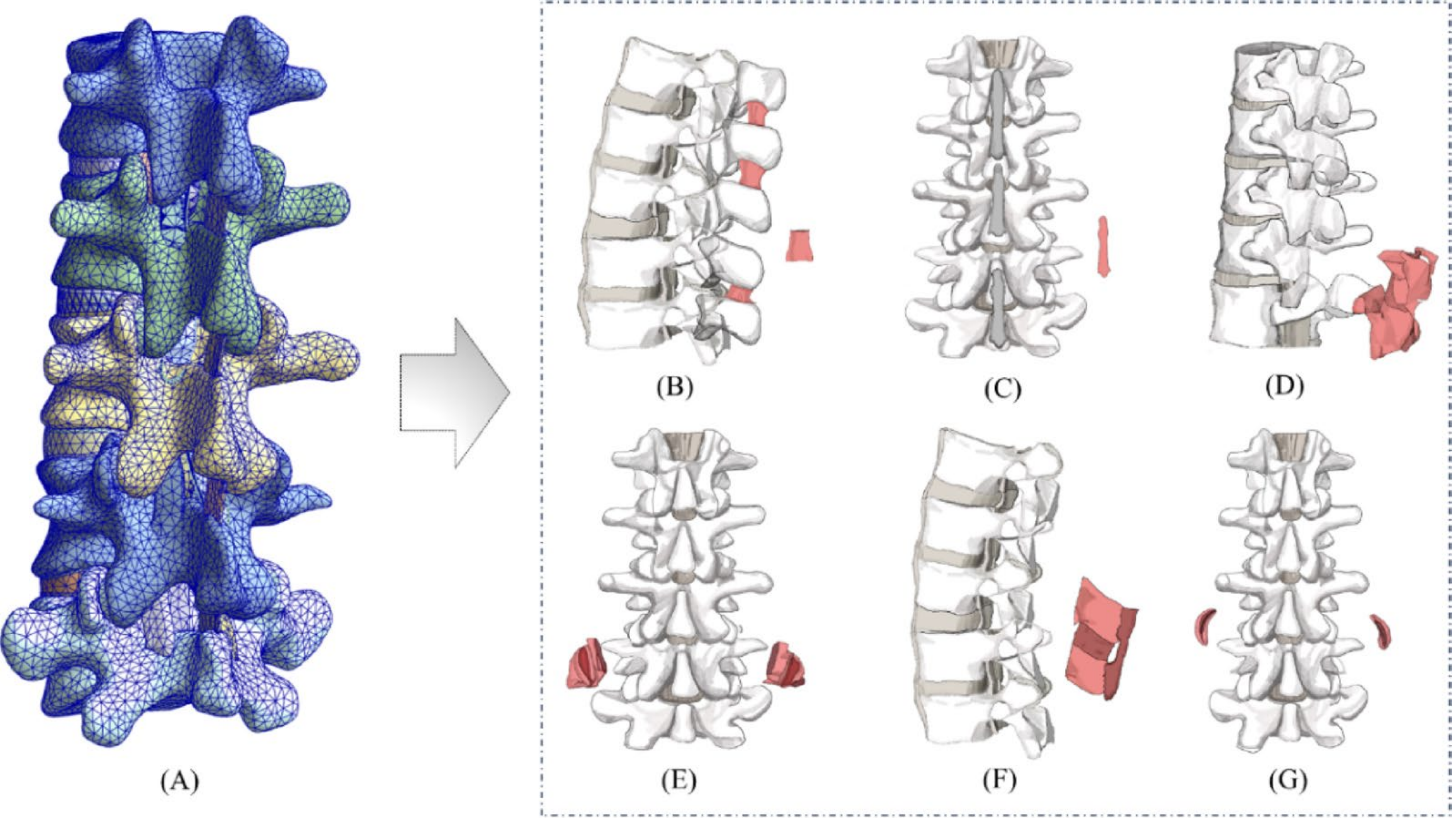
Finite element models of L1-L5 segments and transection of six subsequent steps was performed. (**A**) finite element models, (**B**) interspinous ligament, (**C**) supraspinous ligament, (**D**) ligamentum flavum, (**E**) facet joints, (**F**) inter- and supraspinous ligaments, (**G**) facet joint capsule

#### Combined multi-ligament injury condition

After simulating partial damage to a single ligament, multiple ligaments were progressively resected to create a sequence of injuries, ranging from mild to severe. For each fully resected ligament, its corresponding finite element components were removed from the model, thereby eliminating any biomechanical contribution. The sequence and configuration of multi-ligament injuries were designed according to research objectives and clinical priorities [21]. Specifically, the interspinous ligament was the first to be excised, followed by the ligamentum flavum, and then the facet capsule ligaments and supraspinous ligament were progressively weakened (Fig. 1). This approach enabled for the exploration of the cumulative effects of ligament injuries on spinal stability.

### Meshing and load application

After defining material properties and contact relationships, the model was meshed, resulting in a total of 224,961 nodes and 119,559 elements. Boundary conditions were applied, with the inferior surface of L5 fixed to restrict its movement. The superior surface of L1 was defined as the loading surface, where a vertical axial load of 150 N and pure moments of 10 N·m in all directions were applied. These loads were designed to simulate the forces experienced by the spine during motion. To facilitate subsequent calculations of joint RoM, remote points were incorporated to the model. These points allowed for the application of directional moments, and the solution command stream was imported into the root directory to ensure precise displacement and RoM measurements.

A mesh convergence test was conducted to verify the rationality of the meshing and the stability of the results, ensuring that further refinement would not significantly alter key biomechanical parameters. The outcomes of the mesh convergence testing confirmed that the finite element model developed in this study accurately and reliably simulates spinal biomechanical behavior, providing a solid foundation for subsequent injury analysis and data interpretation.

### Mesh convergence study

To ensure that our stress results were independent of element size, we performed a systematic mesh-refinement study on the intact L4–L5 model under 10 N·m flexion. Five meshes were generated, ranging from approximately 50,000 to 200,000 elements. For each mesh we recorded the peak von Mises stress in the intervertebral disc. The relative difference in stress between successive meshes was computed as

$$\varDelta{\%}=\frac{|{\upsigma}_\text{n}-{\upsigma}_\text{n-1}|}{{\upsigma}_\text{n-1}}\times100{\%}$$

**Fig. 2:**
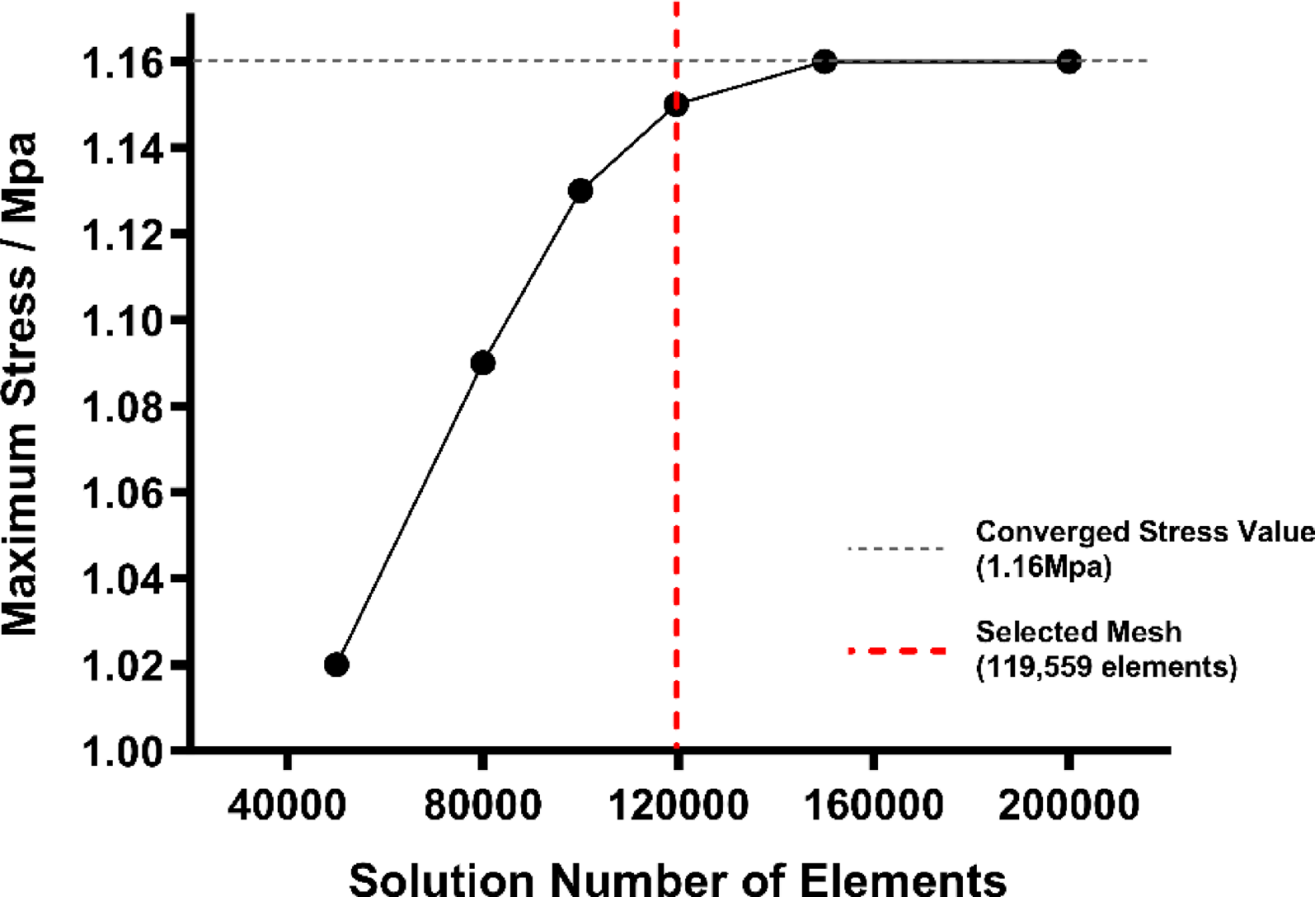
Mesh convergence analysis for the L4-L5 segment under flexion

We adopted the common convergence criterion of < 5% change in stress between two successive refinements. As shown in Fig. 2, the stress plateaued at 1.15 MPa when using 119,559 elements, with a −3.7% change from the 100,000 element mesh. Because this fell below our 5% threshold, we selected the 119,559 element mesh for all subsequent simulations (Fig. 2).

### Validation of model effectiveness

#### Validation of kinematic predictions

**Fig. 3:**
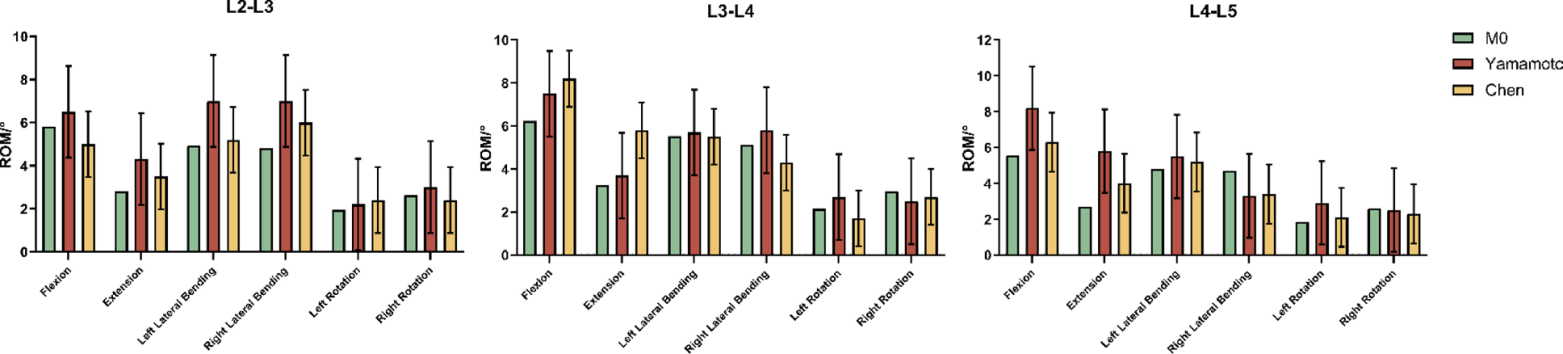
Verification of FE model validity, Comparison of the RoMs with published experimental results

After constructing the L1-L5 finite element model, lumbar spine motion was simulated under six physiological conditions: flexion, extension, left/right lateral bending, and left/right rotation. Regarding the verification of our numerical models, we have ensured that the convergence of the mesh refinement has been appropriately performed. Our studies were compared with biomechanical analysis results from physical and finite element models published by Yamamoto [22], Chen [23] and others. The comparison confirmed that the RoM values for L1-L5 in the developed full model were consistent with those found in the reference literature under these six conditions. For direct comparability with the validation studies (e.g. Yamamoto et al. and Chen et al.), we applied the identical loading and boundary conditions in our FE simulations-namely a 150 N axial preload on the superior endplate and pure bending moments of 10 N·m in flexion/extension, lateral bending, and axial rotation. This validated the model’s simulation results, demonstrating its suitability for this study (Fig. 3).

#### Validation of disc stress predictions

To assess the physiological realism of our finite-element stress predictions, we compared two aspects of the L4-L5 flexion results against in vitro intradiscal pressure (IDP) data reported by Heuer [10].

First, both our finite-element (FE) predicted von Mises stresses and Heuer’s IDP measurements were normalized to the intact (w/o SSL) condition, and the percentage increases were compared following sequential resection of the interspinous ligament (w/o ISL), ligamentum flavum (w/o FL), and facet capsule (w/o FC).

Second, the relationship between von Mises stress and segmental range of motion (RoM) across different PLC injury patterns was analyzed to evaluate the coupling behavior between mobility and disc loading observed experimentally.

### Analysis of segmental RoM and intervertebral disc stress distribution

To evaluate the impact of PLC injuries on the stability of lumbar spinal segments, this study measured both the RoM in various directions and the stress distribution within the intervertebral disc (IVD). Stress data from various components of IVD were extracted using ANSYS software, and changes in segmental RoM were recorded under different ligament injury conditions. These data were used to quantify the biomechanical impact of PLC injuries on spinal stability. Maximum stress values in different regions of IVD were recorded to assess the effect of ligament injuries on stress concentration within the disc. Stress distribution changes were visualized using contour plots and analyzed in conjunction with segmental RoM variations to compare the mechanical responses of the spine under different injury conditions. For each ligament injury pattern (single, double, or combined), the contribution percentage was calculated as the increment in segmental range of motion (RoM) induced by that injury pattern, divided by the total RoM increment observed across all tested patterns within the same category, and then expressed as a percentage. The formula is as follows:

$$\begin{aligned} Contribution_i(\% )=\:&\frac{{RoM_i - RoM_{{\text{intact}}}}}{{\sum\nolimits_{j} {(RoM_j - RoM_{{\text{intact}}})} }} \\ & \times 100\% \end{aligned}$$

where *RoM*_*i*_ is the range of motion for the *i*-th injury pattern, *RoM*_*intact*_ is the RoM of the intact (uninjured) model, and the denominator sums the RoM increments for all patterns within the relevant injury category.

### Statistical analysis

Non-parametric statistical methods were used to evaluate the differences and relationships in the biomechanical indices of lumbar spinal segments under various states of PLC injuries. All data processing and statistical analyses were conducted using the Python programming language (version 3.13.1). The significance level was set at α = 0.05 (two-tailed).

#### Group difference analysis (Kruskal-Wallis test and Dunn Post-hoc test)

The biomechanical data obtained in this study comprise independent, non-normally distributed samples that do not satisfy the assumption of homogeneity of variance. Therefore, the Kruskal-Wallis test was applied to analyze overall differences among multiple groups. When significant differences between injury conditions (ligament combinations) were detected using the Kruskal-Wallis test, Dunn’s post-hoc test was performed to identify the specific sources of these differences.

#### Effect size (η²) analysis

To assess the practical significance of the group differences, the effect size (η²) was calculated for the Kruskal-Wallis test. The effect size was estimated using the following formula:

$$\eta^2=\frac{H-(K-1)}{N-1}$$

Where:

- H is the Kruskal-Wallis test statistic,
- k is the number of groups,
- N is the total sample size.

According to Cohen’s standards, the η² values are categorized into three levels:

- Small: approximately 0.01,
- Medium: approximately 0.06,
- Large: approximately 0.14.

These values were used to assess the biological significance of each ligament’s contribution to the biomechanical stability of the lumbar spinal segment.

## Results

### Validation of disc stress predictions

**Fig. 4:**
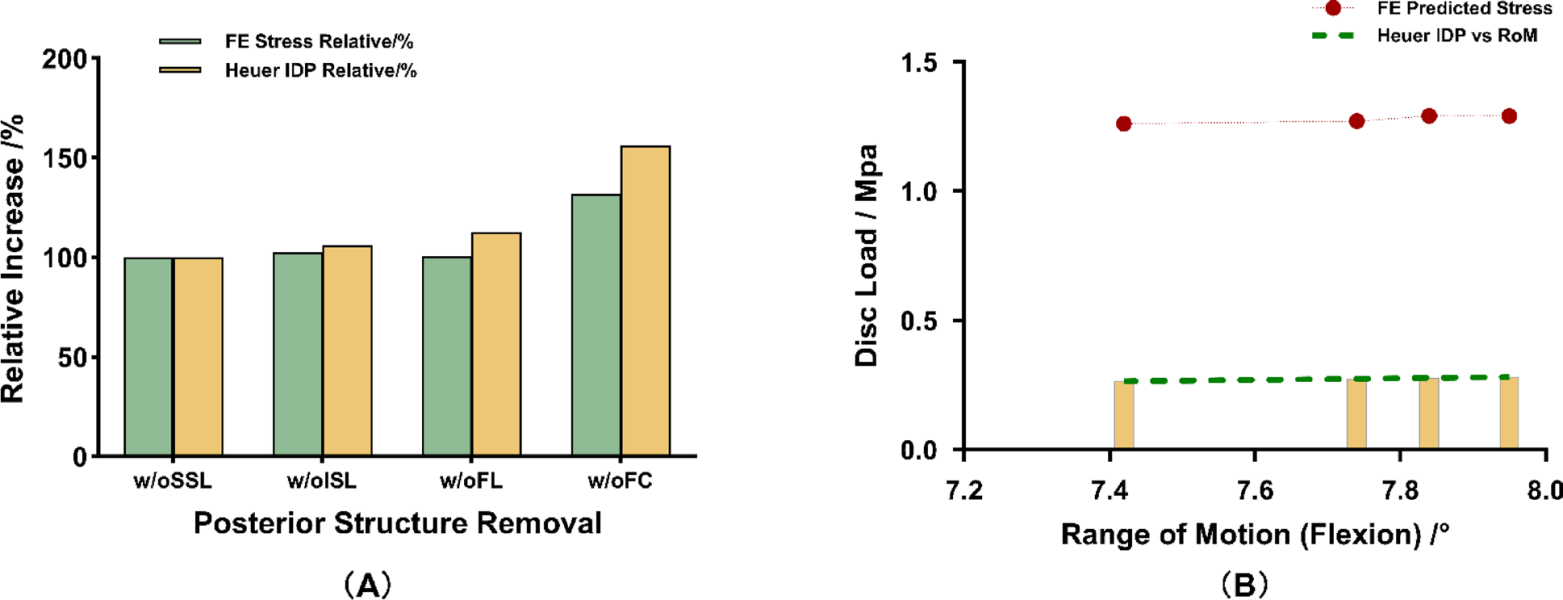
Validation of finite-element predicted disc stresses against experimental intradiscal pressure (IDP) data under sequential posterior ligament removal reported by Heuer et al. [10]. (**A**) Relative increase in disc load (normalized to the w/o SSL baseline) comparing FE model predictions and Heuer experimental measurements under 10 N·m flexion, following sequential resection of ISL, LF, and FC. (**B**) Relationship between disc load and flexion RoM: comparison of FE model scatter and Heuer [21] regression trend after combined PLC injuries

Despite differences in axial preload (500 N in Heuer’s study [10] versus 150 N in our model), the relative rise in disc loading was remarkably similar: our FE model predicted increases of 102.4%, 100.8%, and 132.2%, compared to Heuer’s experimental increases of 106.3%, 112.5%, and 156.3%, respectively (Fig. 4A). This comparison was based on trend consistency, not absolute load equivalence. This strong agreement in trend confirms that our model accurately captures the biomechanical sensitivity of disc load to posterior structure compromise.

Although the relationship appears flat, a general trend of increasing disc stress with RoM was observed, echoing Heuer et al.‘s experimental findings [10] (Fig. 4B). This finding further validates that our model replicates the expected coupling between increased mobility and elevated disc loading following progressive destabilization.

Together, these results demonstrate that our finite-element model faithfully reproduces both the pattern and magnitude trends of disc stress changes after posterior ligamentous complex injuries, despite differences in axial loading conditions.

### Validation of kinematic predictions

**Fig. 5:**
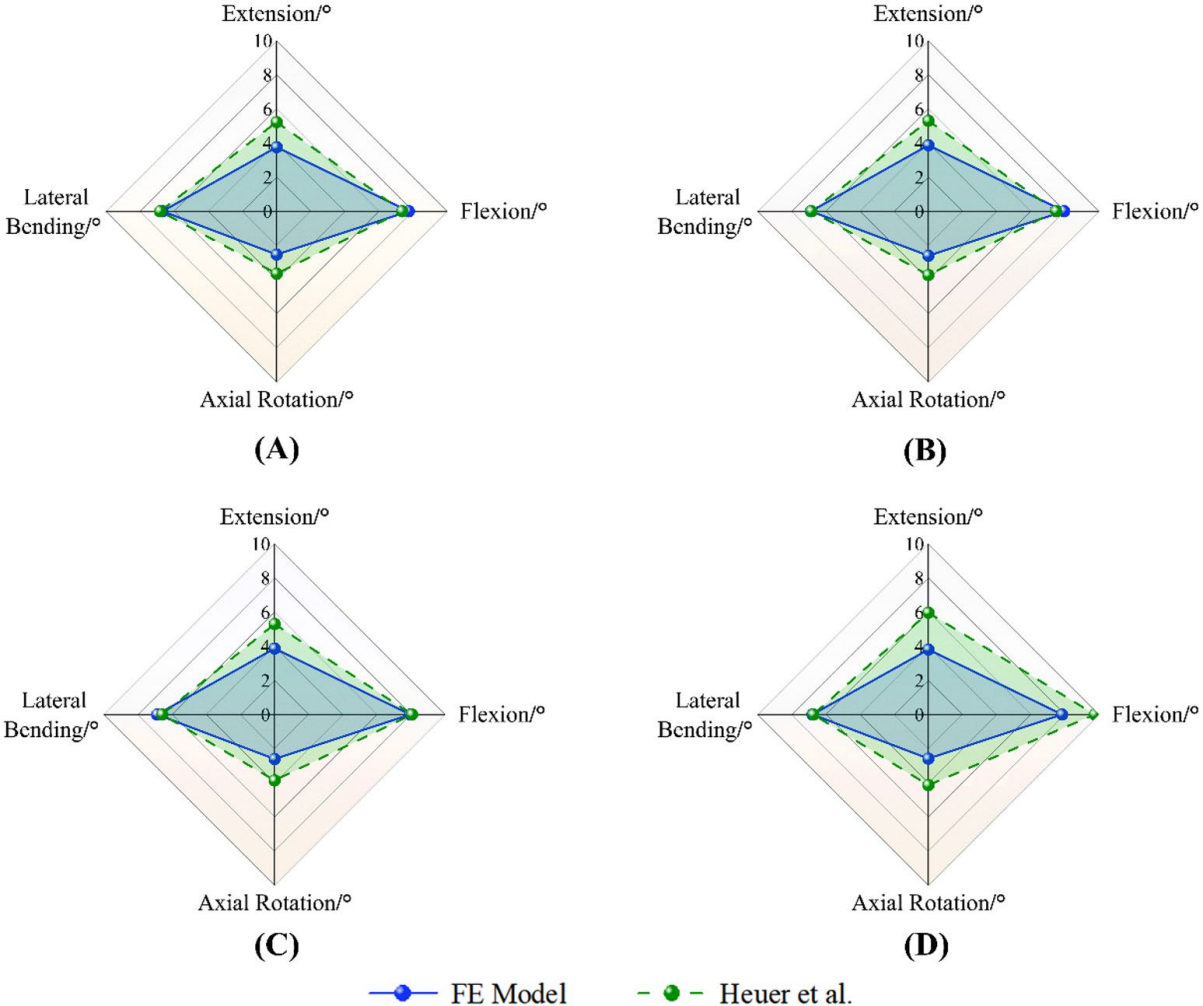
Radar plots comparing finite-element (FE) predicted and experimental (Heuer [21]) RoM changes at L4–L5 across four sequential injury stages. (**A**) w/o SSL (baseline intact model); (**B**) w/o ISL (ISL resected on SSL-removed base); (**C**) w/o FL (ISL + FL resected); (**D**) w/o FC (ISL + FL + FC resected)

To further verify the kinematic accuracy of our FE model, we compared our predicted lumbar segmental RoM with experimental data from Heuer et al. (2007) [21]. Radar plots (Fig. 5) clearly demonstrate the comparison of RoM across four major lumbar motion directions—flexion, extension, lateral bending, and axial rotation—under a 10 N·m moment for each of four sequential PLC injury patterns (w/o SSL, w/o ISL, w/o FL, w/o FC).

The polygon shapes and relative RoM patterns generated by our FE model closely matched those observed experimentally. Notably, both datasets consistently indicated the most substantial increase in RoM following facet capsule resection (w/o FC). Absolute differences in RoM values were primarily due to differing axial preload conditions (150 N vs. 500 N). Nevertheless, the similarity in overall shape and relative magnitude confirms that our FE simulations accurately capture essential biomechanical trends observed experimentally, thereby strengthening confidence in our model predictions.

### Segmental Biomechanical changes and statistical analysis

**Fig. 6:**
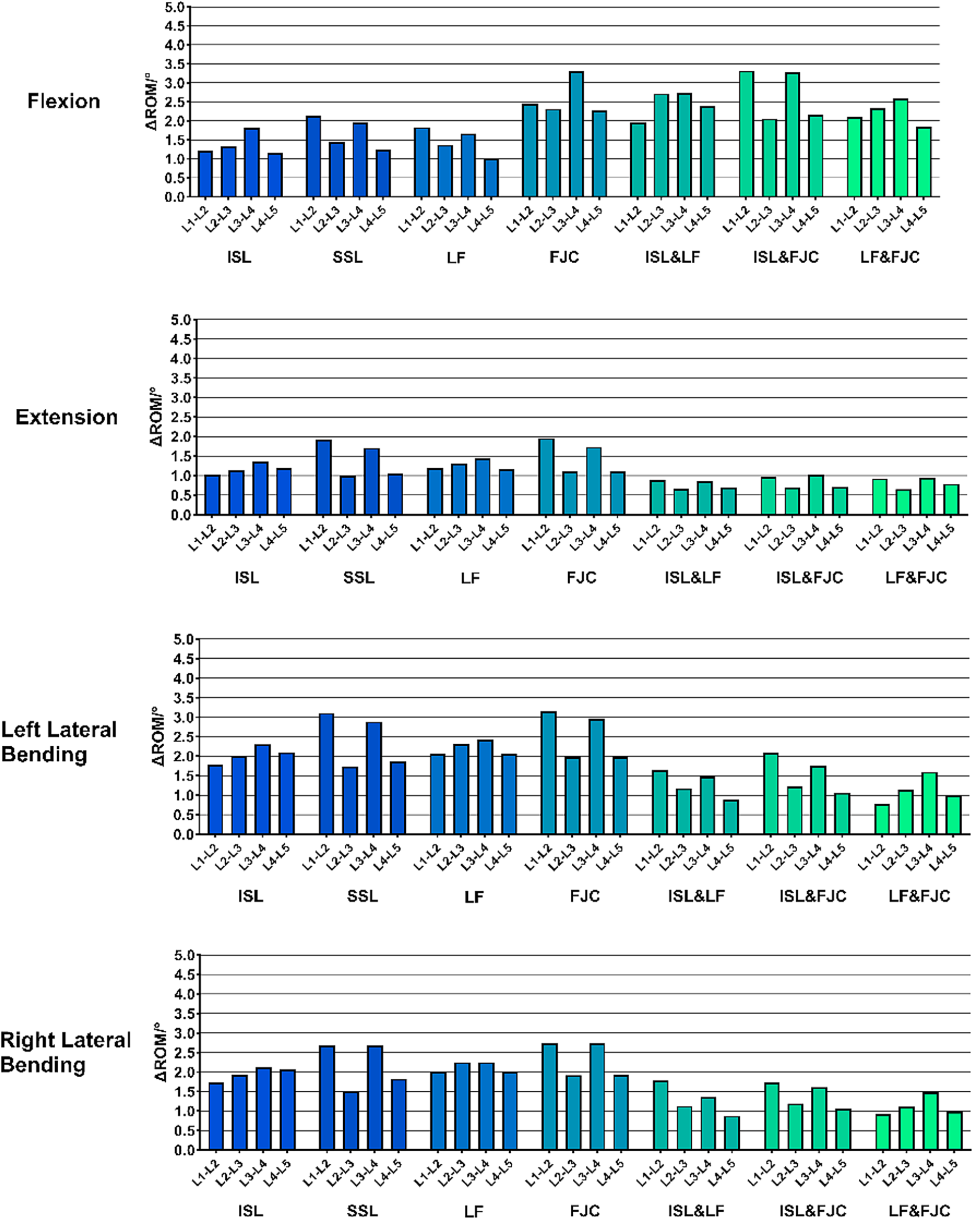

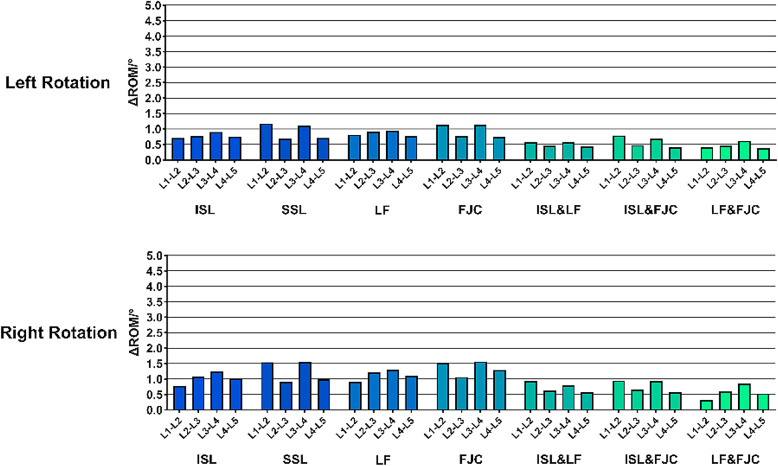
Comparison of L1-L5 segmental mobility under 6 loading conditions across different model groups

This study examined changes in RoM and peak intervertebral disc stress across 12 ligament resection combinations using finite element analysis and experimental measurements (Tables 2 and 3). The Kruskal-Wallis test revealed a significant overall effect of these combinations on lumbar biomechanical indices (*P* = 0.016). However, subsequent multiple comparisons showed a dispersed distribution of differences among the injury patterns, with no significant differences detected between any two specific combinations. To evaluate the practical significance of the observed group effects, the effect size (η²) was calculated, resulting in a value of approximately 0.058. This result suggested that ligament injuries had a moderate impact on segmental stability (Fig. 6).

**Table 2:**
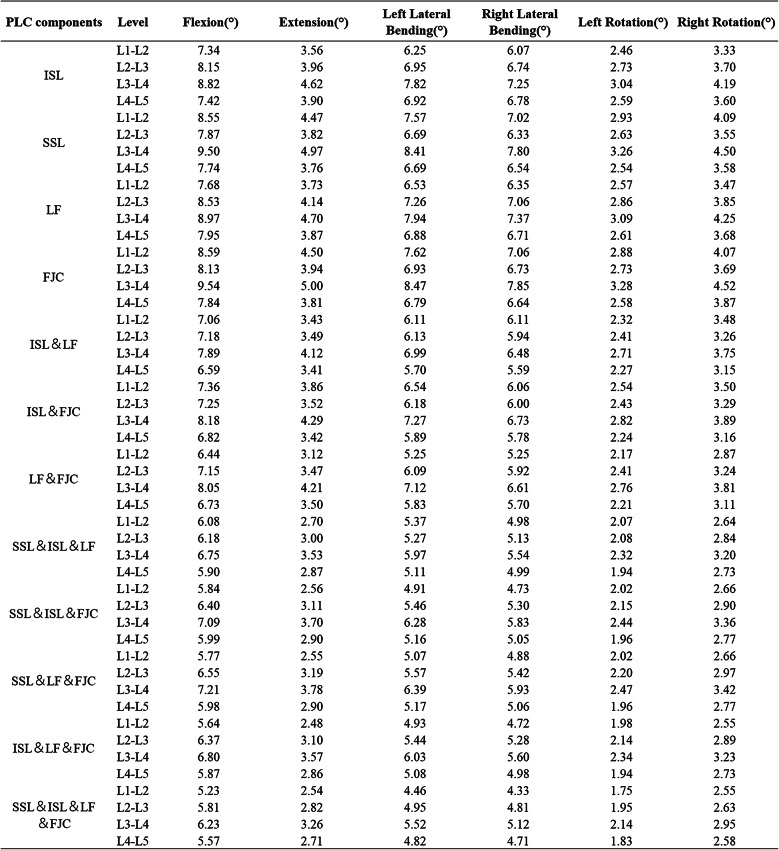
RoM in 12 motion directions of different L1-L5 models

SSL: supraspinal ligament; ISL: interspinous ligament; LF: ligamentum flavum; FJC: facet joint capsule

**Table 3:**
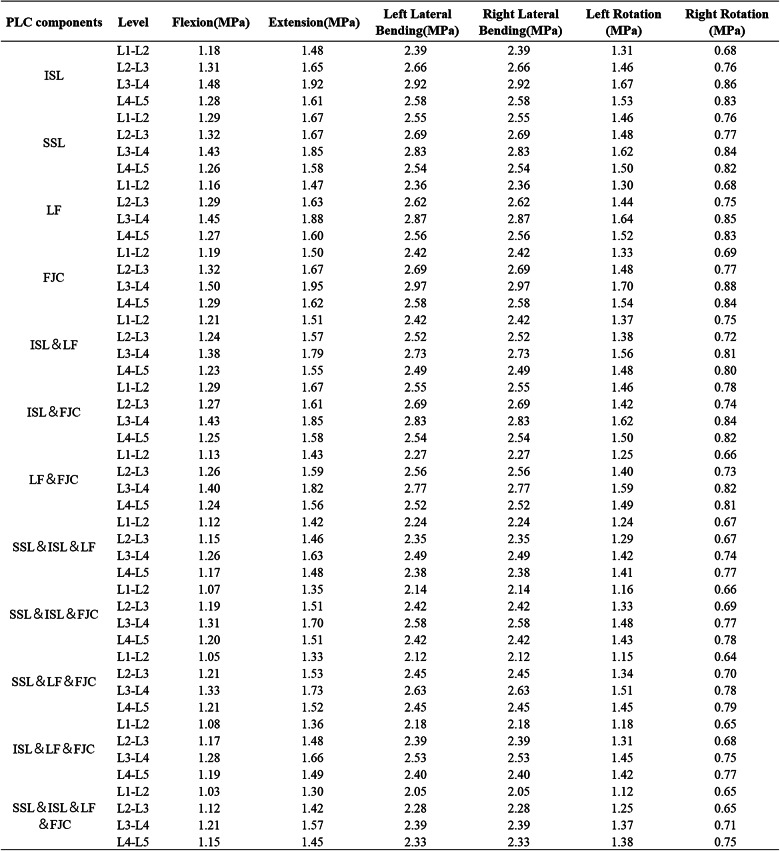
Maximum values of intervertebral disc Von Mises stress in 12 motion directions of different L1-L5 models

SSL: supraspinal ligament; ISL: interspinous ligament; LF: ligamentum flavum; FJC: facet joint capsule

### Influence of single and combined ligament resections on lumbar stability

**Fig. 7:**
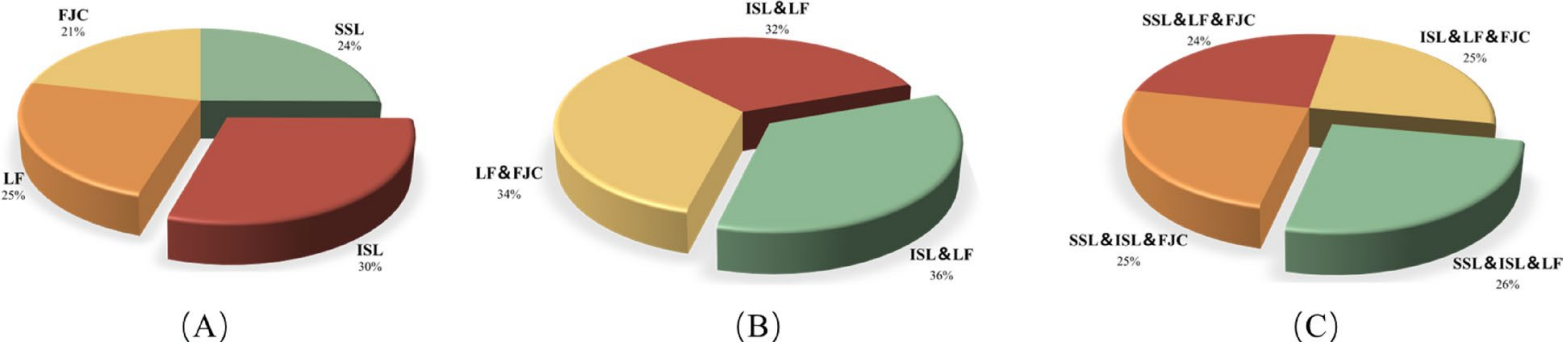
Contribution (%) at different cutting conditions as pie chart. (**A**) Single Ligament Contributions; (**B**) Double Ligament Contributions; (**C**) Combined Ligament Contribution

**Fig. 8:**
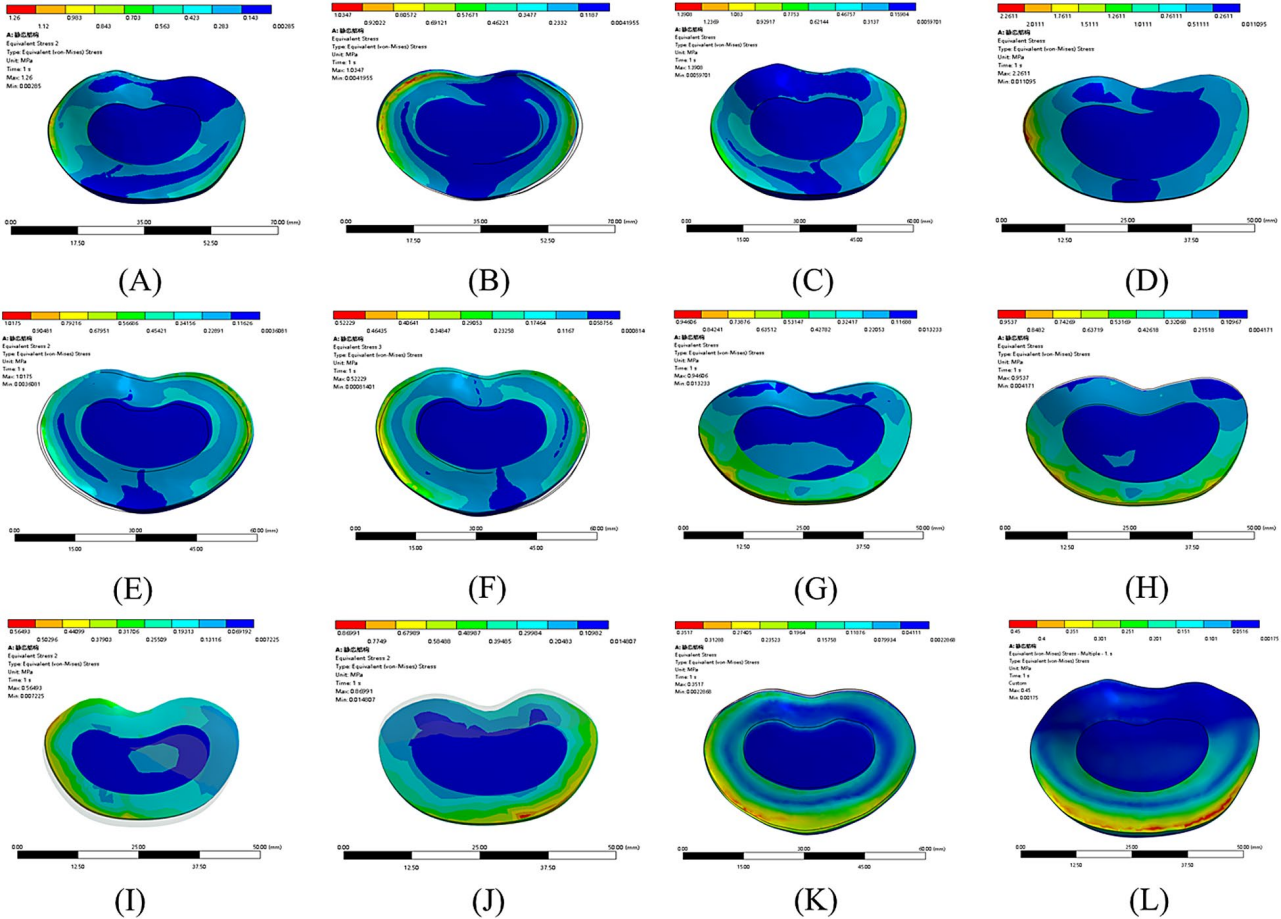
Stress distribution maps of intervertebral discs under 12 different ligament combination injury patterns. (**A**) SSL&ISL&LF&FJC, (**B**) ISL&LF&FJC, (**C**) SSL&LF&FJC, (**D**) SSL&ISL&FJC, (**E**) SSL&ISL&LF, (**F**) LF&FJC, (**G**) ISL&FJC, (**H**) ISL&LF, (**I**) FJC, (**J**) SSL, (**K**) LF, (**L**) ISL

Analysis of the relative contributions of individual ligaments to lumbar stability indicated that the interspinous ligament played the most prominent role in restricting excessive spinal motion and maintaining an appropriate RoM. The ligamentum flavum was the second most significant, while the supraspinous ligament and facet joint capsules contributed to a comparatively lesser extent (Fig. 7). The “contribution (%)” values shown in Figure 7 were calculated as the proportion of the RoM increment induced by each specific ligament resection pattern relative to the total RoM increment observed across all tested patterns within each category. This approach quantifies the relative biomechanical impact of individual and combined ligament injuries on lumbar instability. Analysis of multiple ligament resection combinations showed that combined injury of both the interspinous ligament and ligamentum flavum significantly increased the risk of segmental instability. The joint resection of the facet joint capsules and ligamentum flavum contributed more effectively to spinal stability than the combination of the facet joint capsules and interspinous ligament, followed by the combination of the supraspinous ligament and interspinous ligament. These combinations exhibited a descending order of their contribution to lumbar segmental stability (Fig. 8).

## Discussion

This study used FEA and experimental measurements to explore the relative contributions of different ligaments and their combinations to spinal stability, as well as stress distribution in adjacent intervertebral discs. The study hypothesized that resection of different ligaments would have varying impacts on the biomechanical stability of lumbar segments and stress distribution within intervertebral discs. Furthermore, it was hypothesized that the combined multi-ligament resection would result in synergistic effects, causing a significant decline in stability and stress redistribution, thereby demonstrating the nonlinear cumulative effects of ligament injuries on segmental stability. The results supported these hypotheses, demonstrating that various ligaments within the PLC play distinct yet critical roles in spinal stability. Moreover, in cases of multi-ligament injuries, the remaining intact ligaments provided functional compensation, thereby mitigating the impact of single-ligament injuries on overall stability.

### Comparison with existing literature

Existing research has clearly established the critical role of the interspinous ligament (ISL) in maintaining spinal stability during flexion [4]. This study further confirmed that the ISL has a significantly greater impact on restricting lumbar flexion compared to the ligamentum flavum (LF) and facet joint capsules (FJC), in line with Anderson and Shahidi’s findings that the LF played a secondary role in limiting lumbar flexion [5]. Consistent with the experimental observations of Heuer [21], our study demonstrated incremental increases in disc stress and vertebral range of motion following stepwise resection of the posterior ligamentous complex. Their systematic biomechanical analysis of sequential ligament removal provided robust experimental benchmarks against which our finite-element predictions were validated, confirming the accuracy and clinical relevance of our modeling approach. Moreover, Kettler pointed out that functional spinal unit (FSU) testing often underestimates the role of ligaments, as lumbar RoM is greater in multi-segment column tests [24]. This discrepancy could account for the differences between Adams’s findings and the present study concerning the role of the ISL [25]. While Adams et al. (1980) argued that resistance to flexion is primarily provided by the facet joint capsules and intervertebral discs, this study emphasizes the dominant role of the ISL under high load conditions.

The synergistic amplification effect of multi-ligament injuries on stability was validated in this study. Consistent with Wu [26], the combined resection of the ISL and LF significantly reduced segmental resistance to flexion, highlighting their complementary roles: the ISL primarily limits RoM, while the LF redistributes stress within the intervertebral discs. Zander et al. (2004) further demonstrated that severing the ISL increases the load on the anterior ligamentous complex (ALC), whereas damage to the posterior longitudinal ligament (PLL) or supraspinous ligament (SSL) has a lesser impact on the ALC, indicating the presence of a mechanical compensation network among spinal ligaments [27]. This complementarity is particularly evident during axial rotation. During ipsilateral rotation, the ALC experiences the highest stress, and after ALC injury, stress on the ISL increases significantly. However, the contributions of the LF and SSL remain negligible [9]. Furthermore, the combined injury of the FJC and LF has a greater impact on spinal stability than the combination of FJC and ISL [28]. This observation is consistent with Widmer’s findings, suggesting that the varying damage thresholds of different ligament combinations should be incorporated into clinical risk assessments [3].

The integrity of the PLC is crucial for maintaining postoperative stability. Wei et al. (2023) found that after spinal fusion, both the RoM and von Mises stress in adjacent segments significantly increased, which aligns with the increased risk of adjacent segment disease (ASD) observed in literatures [29]. Huang et al. (2016) further emphasized that preserving the attachment points of the SSL and ISL during surgery can reduce intervertebral disc pressure (IDP) by maintaining the tension-band effect [12]. Compared to total laminectomy, which disrupts the posterior complex, leading to a dramatic increase in IDP and RoM. These findings support the idea of the PLC acting as a “posterior tension band,” as highlighted in previous studies [30, 31], particularly in resisting excessive flexion and subluxation. This mechanism aligns with the Ligament Complementarity Theory proposed by Zander [9]. For instance, damage to the ISL can be partially compensated by enhancing the stability of the FJC, while the functional redundancy of the SSL may delay the progression of ASD. These findings highlight the importance of prioritizing the preservation of the PLC-particularly the ISL and SSL-during surgery. Furthermore, exploring reconstructive techniques, such as re-suturing the thoracolumbar fascia, to restore the tension-band effect is crucial. Nonetheless, careful consideration must be given to the cumulative risks of multi-ligament injuries, particularly the sharp decline in resistance to flexion after the combined resection of the ISL and LF.

Existing research has primarily focused on single-ligament injuries, with limited quantitative analysis of multi-ligament combinations and gradient injuries. This study systematically quantified the contribution of the PLC to spinal stability using effect size analysis (partial η² = 0.058, indicating a moderate effect according to Cohen’s standards). Following the Kruskal-Wallis test, Dunn’s post-hoc analysis revealed no significant pairwise differences, likely due to the small sample size (*n* = 3) and partial mechanical compensation between injury modes. Nevertheless, the moderate effect size (η² = 0.058) suggests clinically meaningful biomechanical variation across groups. While statistical significance was limited, the observed trends support the notion that ligament integrity substantially influences spinal mechanics. This approach provides a more comprehensive understanding compared to the discrete trends observed by Wu et al. (2018) and introduces the effect size model into multi-ligament injury analysis for the first time, thereby establishing the foundation for developing spinal injury prognosis prediction models. Building on Zander’s analysis of the FCL-ISL mechanical network, this methodological framework can be extended to other ligament combinations (such as FCL and LF), providing a robust foundation for developing predictive models of spinal injury prognosis.

### Integration and significance of key findings

Through FEA and experimental measurements, this study elucidated the relative importance of individual ligaments within the PLC in maintaining lumbar spinal stability. The integrity of the PLC is essential for regulating intervertebral micromotion, distributing stress concentrations, and maintaining an appropriate RoM. The results reveal significant variability in the effect of single-ligament injuries on spinal stability. Among the ligaments, damage to the ISL had the most pronounced impact on restricting excessive spinal motion, followed by the LF Injuries to the SSL and FJC had relatively minor impacts. These findings align with previous research, underscoring the critical role of the ISL in limiting excessive flexion and rotation.

This study revealed that combined injury of the ISL and LF had the most significant impact on spinal stability, emphasizing their essential synergistic role. This finding has important clinical implications, particularly in cases involving multiple PLC injuries [32]. Understanding the extent of ligament damage and its biomechanical consequences is crucial for developing surgical strategies that optimize postoperative stability and rehabilitation outcomes.

### Limitations and future directions

FEA provided a precise mechanical simulation tool for this study, although certain some limitations persist. The model simplified the complex dynamic behavior of the spine under actual physiological loads, particularly in motion and non-static conditions, by excluding the contributions of muscles and neural elements [14]. These structures play a critical role in spinal stability and could influence the biomechanical behavior of the spine in real physiological conditions. Additionally, the limited sample size and the inability of the FEA model to account for individual anatomical and biomechanical variations may limit the generalizability of the findings.

Future research should aim to increase the sample size to enhance the statistical power of multiple comparisons and further elucidate the specific effects of different ligament combinations on spinal stability across various motion directions. Validation with clinical data is essential to investigate the biomechanical manifestations of PLC injuries in diverse populations, including different age groups and genders, thus improving the broad applicability of the findings [33].

## Conclusion

This study demonstrated the critical role of the PLC in maintaining spinal stability, particularly the contributions of the interspinous ligament and ligamentum flavum. Our findings further support the association between PLC injuries and spinal instability, offering a quantitative basis for clinical evaluation. Although multiple comparisons did not reveal specific significant differences between groups, the overall effect size and ligament ranking corroborate the hierarchical contributions of individual PLC components to spinal stability. These results not only enhance the theoretical foundation of spinal biomechanics but also provide scientific and quantitative guidance for clinical decision-making in surgical planning and rehabilitation.

## Data Availability

The datasets used during the current study are available from the corresponding author on reasonable request.

## Abbreviations

PLC: Posterior Ligamentous Complex
FEA: Finite Element Analysis
RoM: Range of Motion
ISL: Interspinous Ligament
LF: Ligamentum Flavum
SSL: Supraspinous Ligament
FJC: Facet Joint Capsules
ALL: Anterior Longitudinal Ligament
PLL: Posterior Longitudinal Ligament
TL: Transverse Ligament
CL: Capsular Ligament
